# Discovery and validation of DNA methylation markers for overall survival prognosis in patients with thymic epithelial tumors

**DOI:** 10.1186/s13148-019-0619-z

**Published:** 2019-03-04

**Authors:** Songlin Li, Yuan Yuan, He Xiao, Jiajia Dai, Yunfei Ye, Qin Zhang, Zhimin Zhang, Yuhan Jiang, Jia Luo, Jing Hu, Chuan Chen, Ge Wang

**Affiliations:** 1 Cancer Center, Institute of Surgery Research, Third Affiliated Hospital, Army Medical University (Third Military Medical University), No. 10 Changjiang Zhilu, Yuzhong District, Chongqing, 400042 China; 2 Department of Cardiothoracic Surgery, General Hospital of Xinjiang Military Region, Urumchi, Xinjiang, 830000 Uygur Autonomous Region China; 3 Department of Dermatology, The First Affiliated Hospital of Chengdu Medical College, Chengdu, 610500 China

**Keywords:** Thymic epithelial tumors, DNA methylation, Prognostic model

## Abstract

**Background:**

The current prognosis of thymic epithelial tumors (TETs) is according to the World Health Organization (WHO) histologic classification and the Masaoka staging system. These methods of prognosis have certain limitations in clinical application and there is a need to seek new method for determining the prognosis of patients with TETs. To date, there have been no studies done on the use of DNA methylation biomarkers for prognosis of TETs. The present study was therefore carried out to identify DNA methylation biomarkers that can determine the overall survival in patients with TETs.

**Methods:**

Bioinformatic analysis of TCGA 450 K methylation array data, transcriptome sequencing data, WHO histologic classification and Masaoka staging system was performed to identify differentially expressed methylation sites between thymoma and thymic carcinoma as well as the different DNA methylation sites associated with the overall survival in patients with TETs. Using pyrosequencing, 4 different methylation sites (cg05784862, cg07154254, cg02543462, and cg06288355) were sequenced from tumor tissues of 100 Chinese patients with TETs. A prognostic model for TETs was constructed using these four methylation sites.

**Results:**

The TCGA dataset showed 5155 and 6967 hyper- and hypomethylated CpG sites in type A–B3 group and type C group, respectively, of which 3600 were located within the gene promoter regions. One hundred thirty-four genes were silenced by promoter hypermethylation and 174 mRNAs were upregulated. Analysis of univariate and multivariate Cox regression showed significant association between the methylation levels of 187 sites and the overall survival in patients with TETs. cg05784862(*KSR1*), cg07154254(*ELF3*), cg02543462(*ILRN*), and cg06288355(*RAG1*) were identified as independent prognostic factors for overall survival in patients with TETs after adjusting for Masaoka staging in 100 Chinese patients. The prognostic model which consists of the four abovementioned genes had higher accuracy for predicting the 5-year overall survival in patients with TETs as compared to the Masaoka clinical staging. (Time-dependent ROC analysis AUC 1.000 vs 0.742, *P* = 2.7 × 10^−6^).

**Conclusions:**

The methylation levels of cg05784862(*KSR1*), cg07154254(*ELF3*), cg02543462(*ILRN*), and cg06288355(*RAG1*) sites are associated with the progression of TETs and may serve as new biomarkers for predicting the overall survival in patients with TETs.

**Electronic supplementary material:**

The online version of this article (10.1186/s13148-019-0619-z

## Background

Thymic epithelial tumors (TETs) are rare neoplasms arising from the epithelial cells of the thymus with an incidence of 0.13 per 100,000 person/year in the USA [1]. According to the 2015 World Health Organization (WHO) classification, TETs are divided into thymomas (A, A/B, B1, B2, B3 subtypes) and thymic carcinomas (TCs) based on the tumor cell morphology, degree of atypia, and extent of the thymocyte component [2]. The diagnosis of TETs relies largely on histology supported by immunohistochemistry [3]. Most types A and AB thymomas have low malignant potential, whereas types B1, B2, and B3 thymomas are more aggressive, with B3 thymoma having the greatest tendency for intrathoracic spread. On the contrary, thymic carcinoma is a highly aggressive tumor with frequent lymphatic and hematogenous metastasis [4]. However, its prognostic significance in guiding further treatment is controversial [5].

Surgical resection is considered the potential curative treatment. However, local recurrence or distant metastasis may occur in some patients even after complete resection [6]. Masaoka staging system and WHO classification at diagnosis were reported to be the main prognostic factors for recurrence and survival [7]. Although some genetic profiles have recently been reported in TETs, little is known about their genetic variability and clinical value [8]. Therefore, it is necessary to identify novel molecular biomarkers that improve diagnosis, prognosis, and treatment planning.

Epigenetic alterations such as DNA methylation, histone modification, and loss of genome imprinting play crucial roles in the formation and progression of cancer [9]. Over the past decade, many researchers have demonstrated the presence of aberrant DNA methylation in various types of tumor. As is known, aberrant DNA methylation includes global hypomethylation and regional hypermethylation of which regional hypermethylation is generally associated with gene silencing. However, few studies have investigated DNA methylation in TETs [10]. Furthermore, published data on tumor suppressor genes *MGMT* and *RASSF1A* was not closely related with clinical significance. It is therefore necessary to identify DNA methylation biomarkers that could be used for detection and prognosis in TETs.

In this present study, in order to evaluate the potential of DNA methylation markers in the prognosis of TETs, we compared various methylation profiles of thymoma tissues and thymic carcinomas tissues by analyzing 485,000 CpG markers. We managed to identify a methylation marker panel, and this panel was further validated in 100 TETs tissue samples. The results suggest that DNA methylation sites may be potential biomarkers in the prognosis of TETs.

## Results

### Differentially methylated sites in WHO histological type C thymoma

**Fig. 1:**
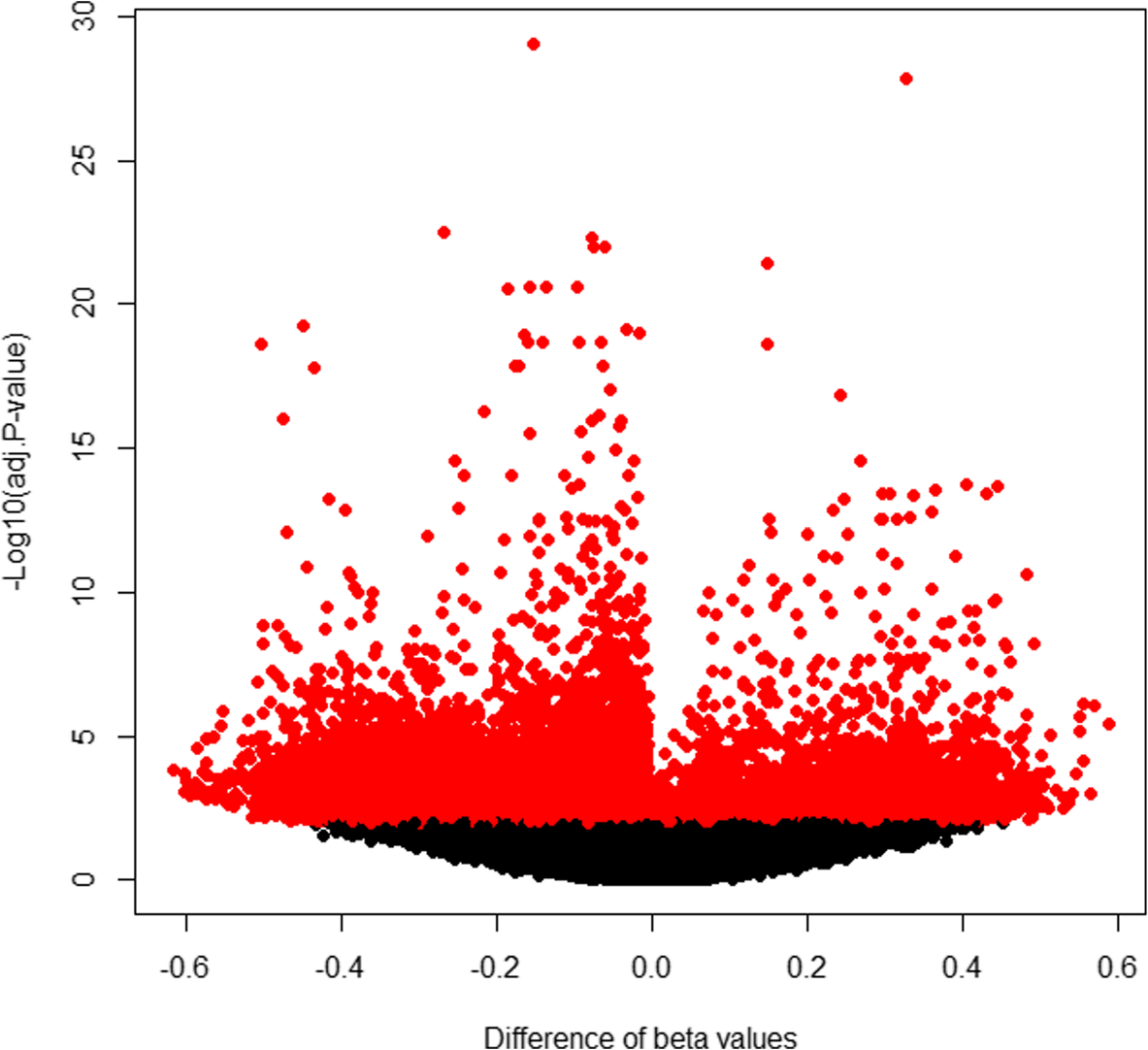
A volcano plots showing significantly expressed methylation sites in 392,653 probes in HumanMethylation450K array between thymoma with WHO histological type C and type A to B3. The red dots represent significantly differential methylation probes among all 392,653 probes included into analysis according to criteria mentioned in the “Methods” section

To identify potential differentially methylated sites specific to WHO histological type C thymoma, DNA methylation profiles of 124 tumor tissues consisting of 113 type A–B3 and 11 type C were used. The clinicopathologic characteristics of these 124 cases are shown in Table 1. A total number of 12,122 CpG sites among 392,653 probes were identified as differentially methylated sites through site level analysis (Fig. 1 and Additional file 1: Table S2), of which, 5155 CpG sites were found to be hypomethylated and 6967 CpG sites hypermethylated. This corresponded to 2693 and 1734 genes respectively. Kyoto Encyclopedia of Genes and Genomes (KEGG) functional enrichment analysis was used to annotate the two set of genes (Additional file 2: Table S3). The genes associated with hypomethylated sites mainly participated in focal adhesion (hsa04510, adjusted *P* = 4.21 × 10^−5^) and apoptosis (hsa04210, adjusted *P* = 2.40 × 10^−4^). On the other hand, the genes regulated by hypermethylation sites manifested more diverse functions and were predominately involved in neuroactive ligand-receptor interactions (hsa04080, adjusted *P* = 4.53 × 10^−24^), calcium signaling pathways (hsa04020, adjusted *P* = 4.72 × 10^−13^), and cAMP signaling pathways (hsa04024, adjusted *P* = 8.33 × 10^−8^).

**Table 1:** Clinicopathologic characteristics of 124 cases from TCGA dataset THYM

Clinicopathologic characteristics		*n* (%)
Overall survival	Non-sensored	114 (92.7)
	Censored	9 (7.3)
Recurrence-free survival	Non-sensored	109 (92.4)
	Censored	9 (7.6)
Gender	Female	60 (48.4)
	Male	64 (51.6)
WHO histological types	A–B3 type	113 (91.1)
	C type	11 (8.9)
History myasthenia gravis	No	87 (71.9)
	Yes	34 (28.1)
Masaoka stage	I–IIB	99 (81.1)
	III–IV	23 (18.9)
Tumor tissue site	Thymus	97 (78.2)
	Anterior mediastinum	27 (21.8)
History of neoadjuvant treatment	No	122 (98.4)
	Yes	2 (1.6)
Postoperative radiotherapy and chemotherapy	No	114 (92.7)
	Yes	9 (7.3)
Radiation therapy	No	80 (65.0)
	Yes	43 (35.0)

### Identification of genes potentially affected by differentially methylated sites in promoter regions

**Fig. 2:**
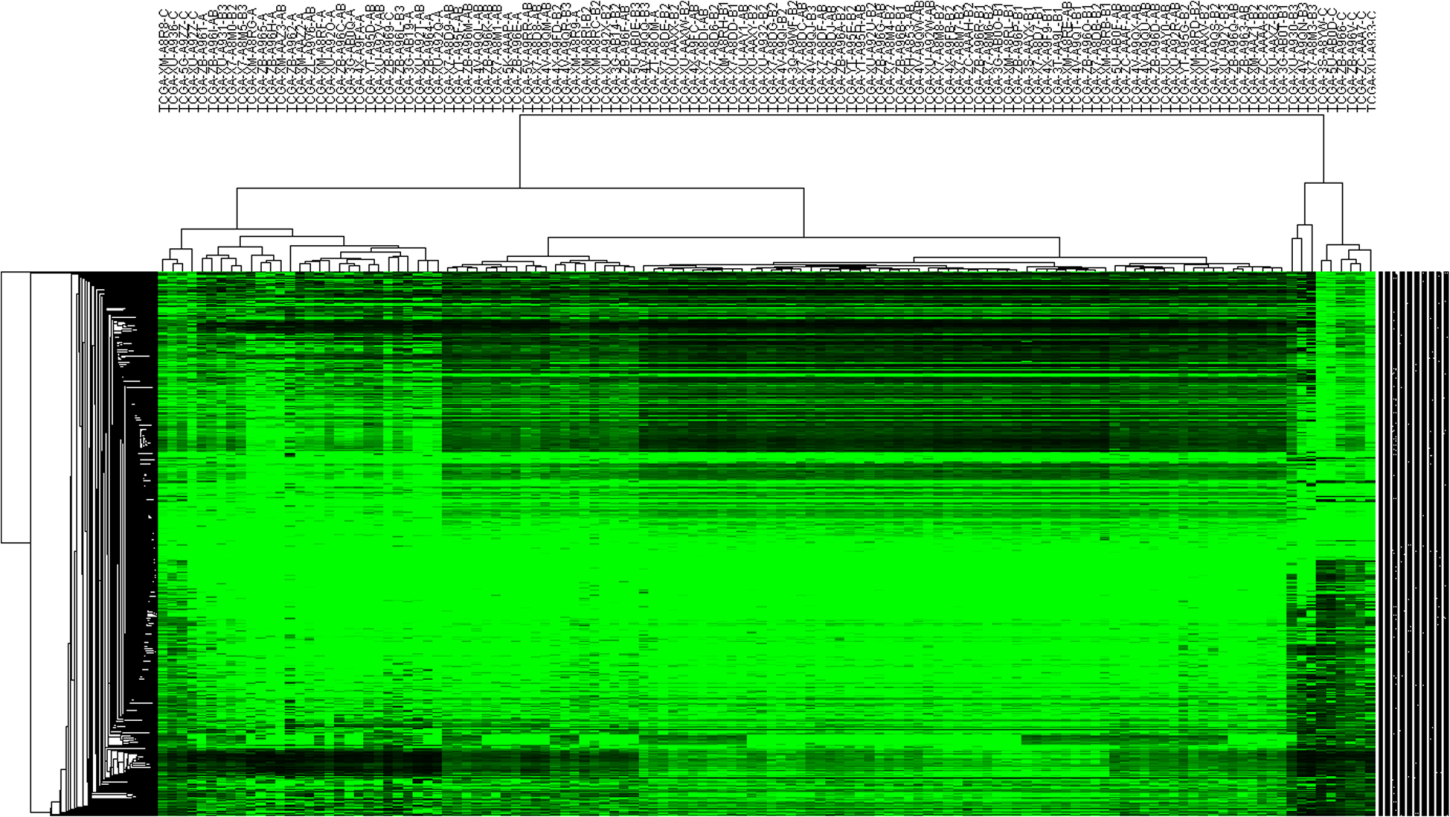
A heatmap showing methylation profiles of 542 significantly expressed methylation sites which localize within promotor regions in corresponding genes and could be involved in regulation of mRNA expression for genes across all 124 cases. The top is the list of patients’ identifiers provided by TCGA, and the terminal characters in each patient’s IDs indicate classification of WHO histological types

Among 12,122 differentially methylated sites, 3600 CpG sites were localized within the promotor regions which corresponded to a total number of 2029 genes which could be transcriptionally regulated. In addition, 3490 genes were identified as differentially expressed genes (DEGs) between WHO histological type A–B3 and type C thymoma through linear regression embedded in limma. However, only 480 genes out of the 3490 DEGs were found to overlap with 2029 genes that had differentially methylated CpG sites in their promotor regions. Based on the criteria proposed under the “Methods” section, 134 genes and 174 genes which corresponded to 268 and 274 CpG sites in their promoter regions were considered epigenetically silenced and upregulated respectively (Additional file 3: Table S4). The heatmap constructed on beta values of these CpG sites across all 124 patients was shown in Fig. 2. This unsupervised cluster analysis revealed three distinct groups of thymoma tumors. It was noteworthy that not all WHO histological type C thymoma are clustered together, and there were some cases where type B3, type AB, and type C thymoma were categorized into one group. Furthermore, the same methylation probes provided similar results in the subset which consisted of type A to B3 thymoma tumors (Additional file 4: Figure S1). These results suggest that thymoma patients diagnosed as the same WHO histological type could exhibit heterogeneity according to methylation profiles.

### Candidate methylation sites for prognosis

**Fig. 3:**
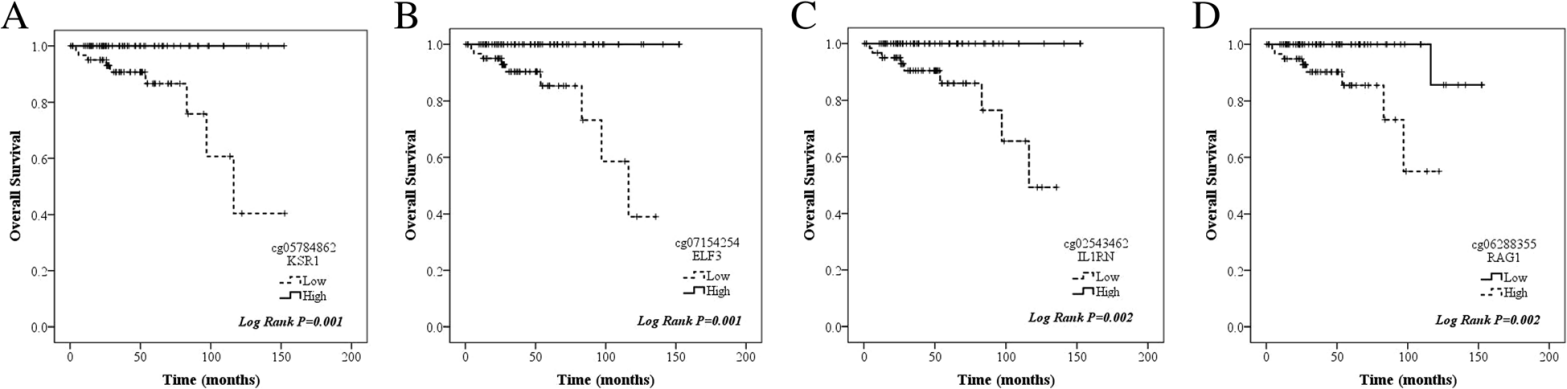
Kaplan-Meier curves showing methylation profile stratifies thymoma patients in whole population into survival subgroups in TCGA dataset. **a**–**c** High methylation in cg05784862(*KSR1*), cg07154254(*ELF3*), and cg02543462(*ILRN*) is associated with significantly longer overall survival. **d** Low methylation in cg06288355(*RAG1*) is associated with significantly longer overall survival

Out of 542 methylation sites which could transcriptionally regulate expression of corresponding genes, 187 CpG sites were identified as potential DNA methylation biomarkers for overall survival in thymoma patients using univariate and multivariate Cox regression (Additional file 5: Table S5). Among these CpG sites, methylation status in four genes, *KSR1*, *ELF3*, *ILRN*, *RAG1*, had shown strong association with overall survival and corresponding mRNA expression (Additional file 6: Figure S2 and Additional file 7: Figure S3). Median beta values for the probes of cg05784862(*KSR1*), cg07154254(*ELF3*), cg02543462(*ILRN*), and cg06288355(*RAG1*) were chosen as cut-off values to categorize patients into low and high methylation subgroups. As shown in Fig. 3, patients with high methylation in the first three methylation sites exhibited excellent prognosis, whereas those with low methylation in the last methylation site were associated with significantly longer overall survival. Moreover, after adjustment for age, gender, WHO histological type, Masaoka stage, presence of myasthenia gravis, tumor site, and radiotherapy, cg07154254(*ELF3*) (HR = 1.091 × 10^−6^ 95% CI 0.000–0.098, *P* = 0.018) and cg02543462(*ILRN*) (HR = 9.744 × 10^−4^ 95% CI 0.000–0.669, *P* = 0.037) remained significantly associated with overall survival. Cg05784862(*KSR1*) (HR = 0.014 95% CI 0.000–1.297, *P* = 0.065) and cg06288355(*RAG1*) (HR = 62.037 95% CI 0.934–4122.5, *P* = 0.054) had borderline significance for overall survival after adjustment mainly because of extremely low number of deaths. These four methylation sites were thus selected as candidates for further validation.

### Validation of these four methylation sites for prognosis in our cohort

**Fig. 4:**
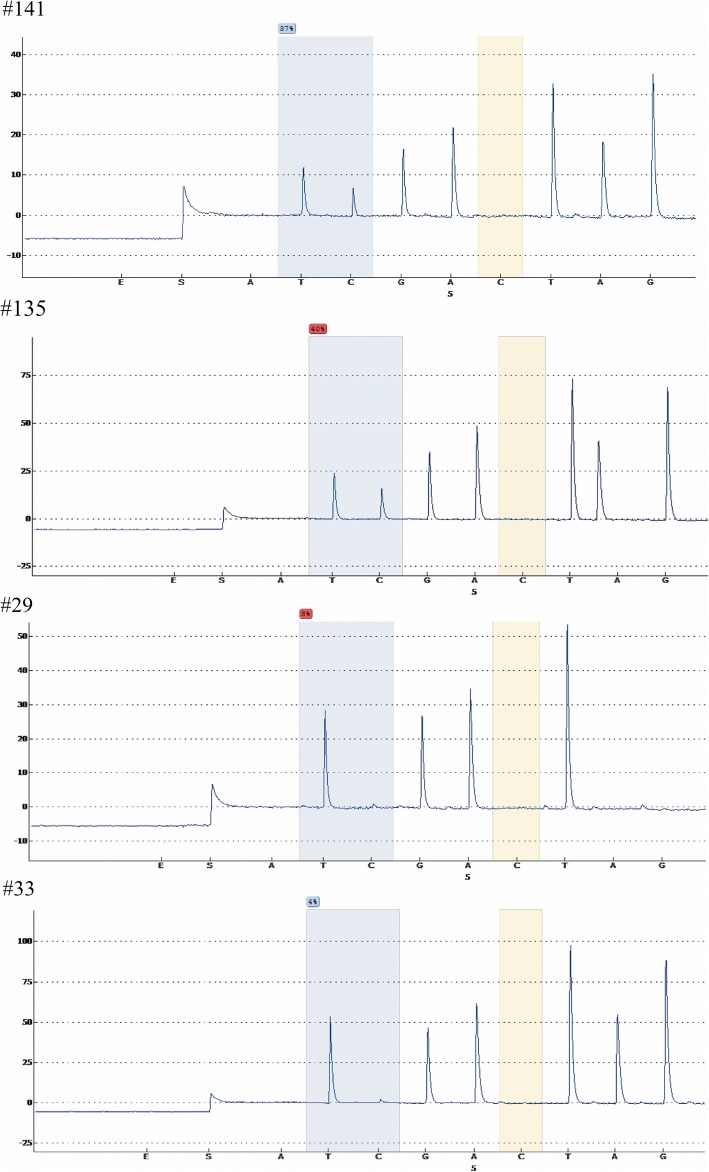
Representative images of pyrosequencing for cg07154254 in *ELF3* in four patients. Increased methylation shown in patients no. 141 and no. 135 and low methylation in patients no. 29 and No.33

**Fig. 5:**
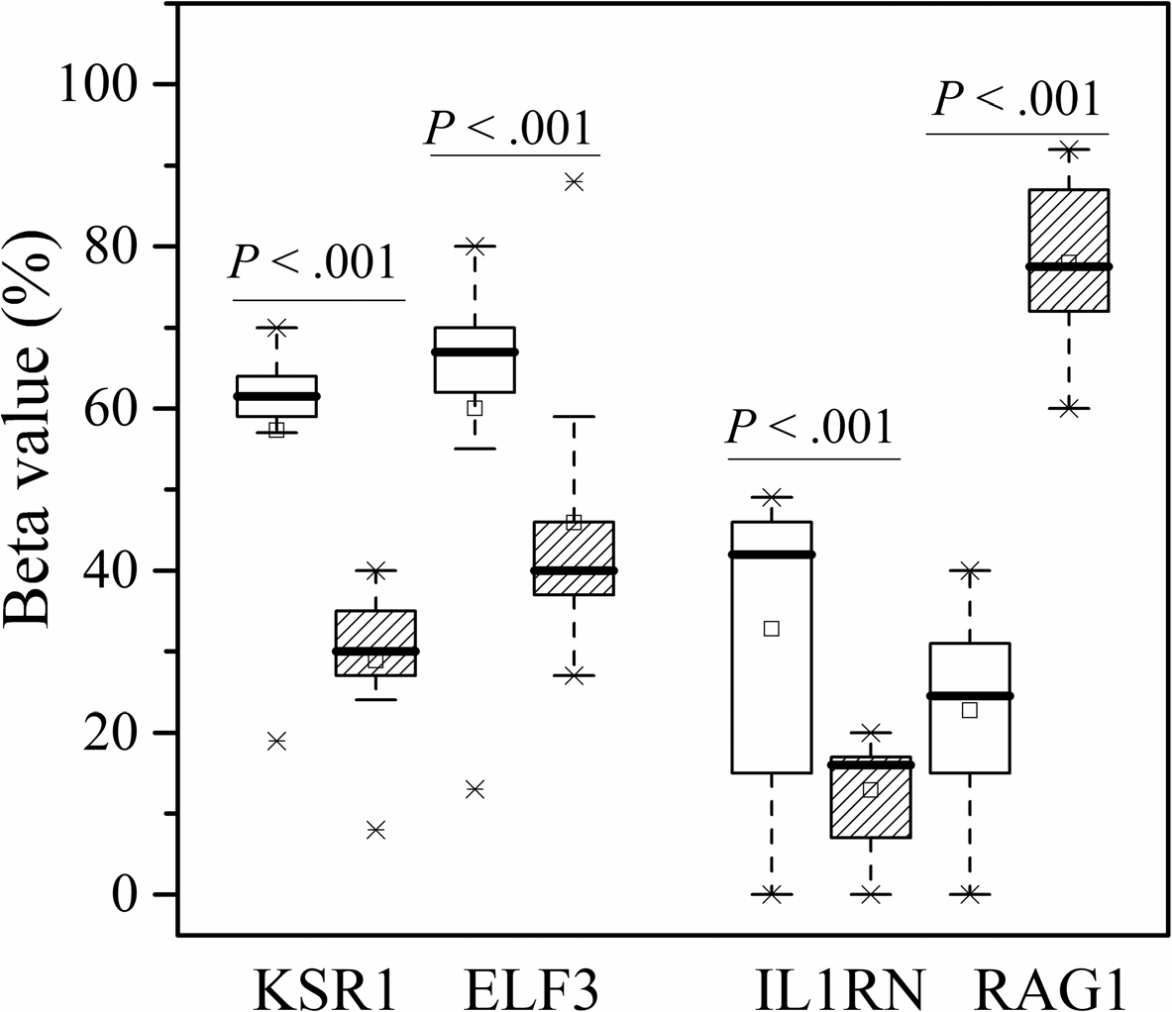
Box plots showing the distribution of beta values in cg05784862 in *KSR1*, cg07154254(*ELF3*), cg02543462(*ILRN*), and cg06288355(*RAG1*) between thymoma with WHO histological type C and type A to B3 in validation set. The boxes with shadow represent patients with WHO histological type C

**Fig. 6:**
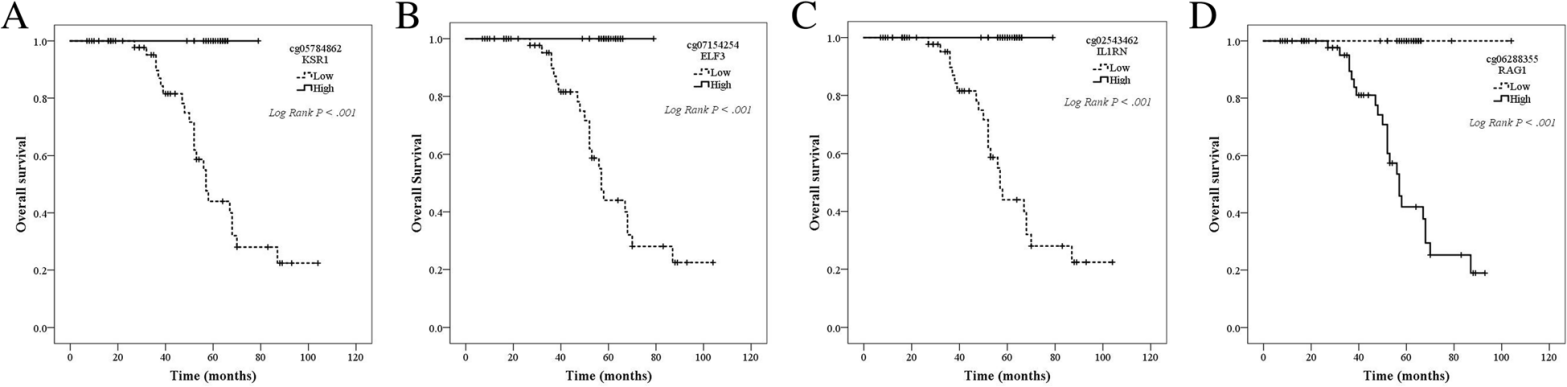
Kaplan-Meier curves showing methylation profile stratifies thymoma patients in whole population into survival subgroups in validation set. **a**–**c** High methylation in cg05784862(*KSR1*), cg07154254(*ELF3*), and cg02543462(*ILRN*) is associated with significantly longer overall survival. **d** Low methylation in cg06288355(*RAG1*) is associated with significantly longer overall survival

**Fig. 7:**
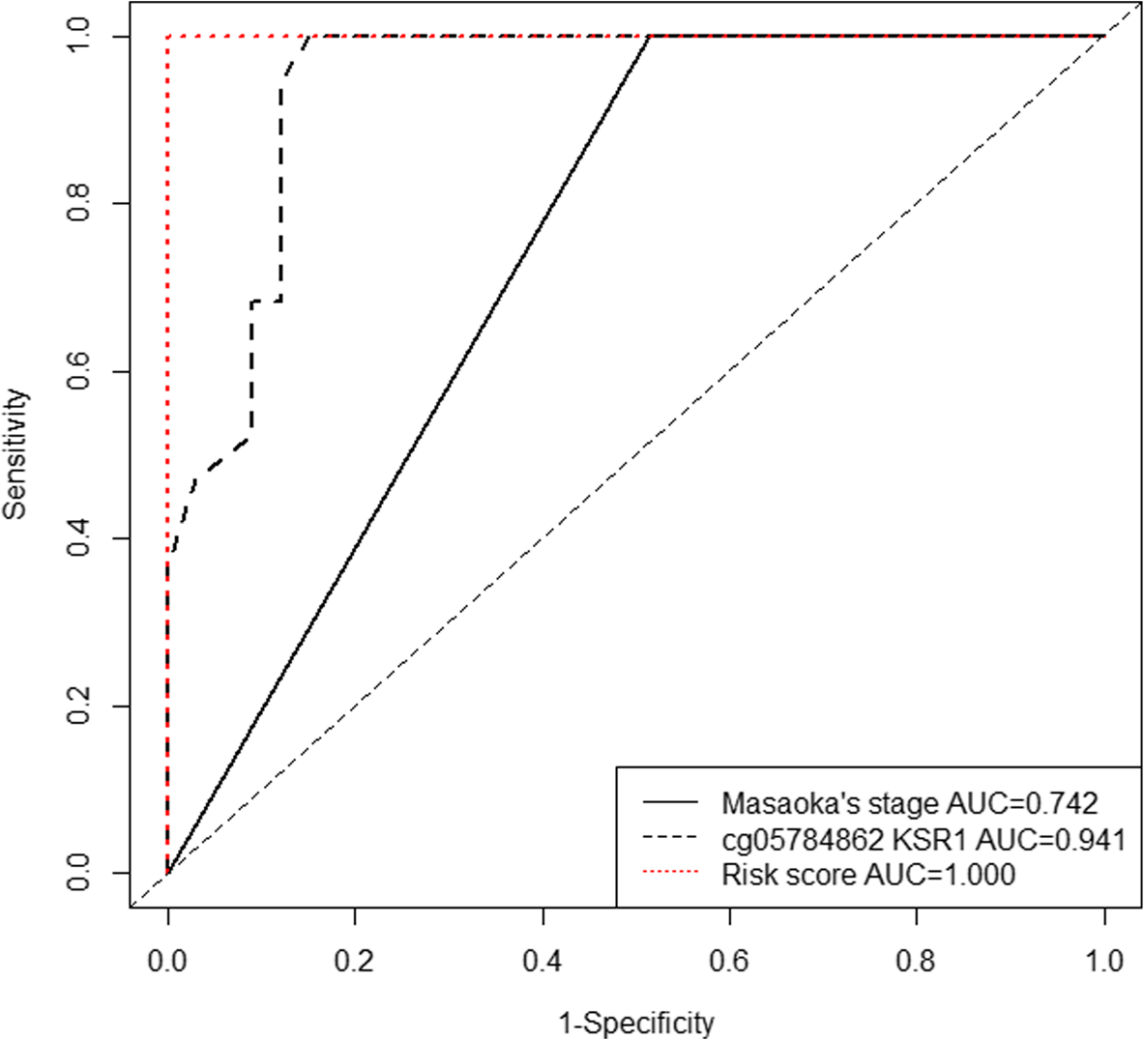
Time-dependent curves showing different capacities for predicting 5-year overall survival in validation set. Risk score is constructed from linear combination of each coefficient in univariate Cox regression for the four methylation sites in TCGA dataset and beta value in validation set as proposed in the “Methods” section

The clinicopathologic characteristics of 100 cases enrolled in validation set are shown in Table 2. Twenty-four deaths were observed during follow-up. In addition, the percentage of patients with WHO histological type C and Masaoka stage III–IV was 46% and 63% respectively, which was significantly higher than those in the TCGA dataset. The representative results of pyrosequencing for methylation status in four patients were shown in Fig. 4. In the whole population, the methylation status in these four candidate methylation sites was revealed to be consistent with the TCGA cohort. Beta values for cg05784862(*KSR1*), cg07154254(*ELF3*), and cg02543462(*ILRN*) were significantly lower in patients with WHO histological type C compared with those with type A–B3. Cg06288355(*RAG1*) exhibited opposite pattern (Fig. 5). Masaoka staging was an independent prognostic factor for overall survival, resulting from forward stepwise Cox regression using age, gender, WHO histological type, Masaoka stage, presence of myasthenia gravis, radiotherapy, and chemotherapy as candidate variables. After adjusting for Masaoka stage, methylation status in the four sites were significantly associated with overall survival (Table 3, Fig. 6), which was consistent with results from the TCGA dataset. Time-dependent ROC curve analysis revealed that risk score from combination of beta values in the four methylation sites was superior to Masaoka stage for prediction of 5-year overall survival (AUC 1.000 vs 0.742, *P* = 2.7 × 10^− 6^) (Table 4, Fig. 7).

**Table 2:** Clinicopathologic characteristics of 100 cases enrolled for validation

Clinicopathologic characteristics		*n*
Survival	Survival	76
	Death	24
Gender	Female	43
	Male	57
Histology	Tumor	54
	Carcinoma	46
Myasthenia gravis	No	69
	Yes	31
WHO histological types	A/AB/B1/B2/B3	54
	C	46
Masaoka stage	I–II	37
	III–IV	63
Radiotherapy	No	66
	Yes	34
Chemotherapy	No	69
	Yes	31
Beta value	WHO histological types A–B3 (median/range)	WHO histological type C (median/range)
cg05784862 KSR1	61.5 (19–70)	42 (0–49)
cg07154254 ELF3	30 (8–40)	16 (0–20)
cg02543462 IL1RN	67 (13–80)	24.5 (0–40)
cg06288355 RAG1	40 (27–88)	77.5 (60–92)

**Table 3:** Results from Cox regression in different populations†

	Univariable Cox regression in whole population		Whole population, adjusted for Masaoka stage		WHO histological type C only, adjusted for age, gender, radiotherapy, and chemotherapy	
	HR (95% CI)	*P*	HR (95% CI)	*P*	HR (95% CI)	*P*
cg05784862 KSR1†	0.868 (0.814–0.926)	< 0.001	0.870 (0.814–0.931)	< 0.001	0.852 (0.768–0.945)	0.003
cg07154254 ELF3†	0.718 (0.611–0.844)	< 0.001	0.720 (0.611–0.848)	< 0.001	0.674 (0.519–0.874)	0.003
cg02543462 IL1RN†	0.868 (0.819–0.920)	< 0.001	0.870 (0.819–0.923)	< 0.001	0.859 (0.798–0.925)	< 0.001
cg06288355 RAG1†	1.202 (1.089–1.328)	< 0.001	1.201 (1.085–1.328)	< 0.001	1.201 (1.083–1.332)	0.001

†All beta values in each methylation sites were entered into equation as continuous variables

**Table 4:** Area under the curves and corresponding 95% confidential intervals predictive for 5-year overall survival†

	AUC 95% CI	Crude *P* compared with Masaoka stage	Adjusted *P* value
Masaoka stage	0.742 (0.656–0.828)		
cg05784862 KSR1†	0.941 (0.881–1.000)	7.63 × 10^−6^	1.526 × 10^−5^
cg07154254 ELF3†	0.940 (0.880–0.999)	7.50 × 10^−6^	2.249 × 10^−5^
cg02543462 IL1RN†	0.966 (0.923–1.000)	2.77 × 10^−7^	1.385 × 10^−6^
cg06288355 RAG1†	0.933 (0.869–0.996)	1.75 × 10^−5^	1.749 × 10^−5^
Risk score	1.000 (0.998–1.000)	6.75 × 10^− 7^	2.7 × 10^−6^

†Masaoka stage was tested as binary variable (III–IV and I–II) and methylation status and risk score continuous variables

More importantly, in the subset that comprised of only WHO histological type C, each of the individual CpG sites was an independent prognostic factor for overall survival after adjusting for age, gender, radiotherapy, and chemotherapy (Table 3). However, it was difficult to draw meaningful results from Cox regression with regards to prognosis of these four methylation sites in the subset of WHO histological type A to B3 because only six patients died.

## Discussion

Thymic epithelial tumors are a group of rare thoracic cancers including thymomas and thymic carcinomas which originate from thymic epithelial cells. Due to the relatively low incidence rate, there is a lack of studies on TETs and therefore little is known about the pathogenesis of TETs [11]. The current prognosis for patients with TETs mainly depends on the WHO histologic classification and the Masaoka staging system which have certain limitations in clinical application. Thus, there is a need for new biomarkers to better improve patient prognosis [12, 13]. Several other studies had identified DNA methylation gene as prognostic biomarkers of nasopharyngeal carcinoma and acute myeloid leukemia [14, 15]. In the current field of TETs, tumor size, gene mutation, protein expression, and miRNA had been reported to affect the prognosis of patients with TETs and potentially be used as prognostic biomarkers for TETs [15, 16]. However, targeting DNA methylation sites as prognostic biomarkers for TETs have not been explored, and our current study is the first to report DNA methylation sites as biomarkers for the prognosis of patients with TETs. In addition, our study also has the highest number of patients enrolled for the prognostic study of patients with TETs, with 124 patient data from the TCGA data portal and100 patient data from our own study.

In this study, potential prognostic biomarkers of TETs were found in sites of DNA methylation in tumor tissues of patients with TETs. Four DNA methylation sites (cg05784862, cg07154254, cg02543462, and cg06288355) that predict the overall survival in patients with TETs were selected, and a prognostic model was constructed which has a higher accuracy compared to the commonly used Masaoka staging (AUC 1.000 vs 0.742, *P* = 2.7 × 10^−6^). This prognostic model could serve as a new method in the clinical field for the prediction of overall survival in patients with TETs.

Besides predicting the overall survival in patients with TETs, the DNA methylation sites were also found to differentiate type C TETs from other types of TETs. Currently, diagnosis of TETs requires pathological study of biopsies [17] and WHO classification by cell morphology which the precision of these diagnosis methods needs to be improved. The four DNA methylation sites (cg05784862, cg07154254, cg02543462, and cg06288355) were selected by comparing WHO’s type A–B3 and type C DNA methylation levels; therefore, they can be used to differentially diagnose for type C TETs. It is important to know that the treatment of type C TETs is very different from type A–B3 [18]; therefore, it is necessary to improve the precision for treatment in different types of TETs.

*KSR1* gene is an oncogene regulated by the cg05784862 site. In this study, patients with poor prognosis showed lower levels of cg05784862 site methylation in the tissue, resulting in higher expression of *KSR1* gene. A recent study by Steven PD et al. [19] reported that suppression of *KSR1* gene expression could prolong survival in patients with colorectal cancer, and McCall JL et al. [20] reported that the *KSR1* gene affects Myc protein expression which affects the prognosis of patients with colon cancer. In addition, Neilsen BL [21] reported that *KSR1* could be a therapeutic target for Ras-dependent cancers. With all the studies done on *KSR1*, this is the first time *KSR1* has been reported to affect the prognosis of patients with TETs. *ELF3*, a transcription factor, is regulated by the cg07154254 site [22]. Our results showed that patients with poor prognosis had lower levels of cg07154254 site methylation in the tissue, which results in higher expression of *ELF3* gene. *ELF3* gene plays a major role in tumor proliferation, invasion, and metastasis. A recent study had reported that activation of *ELF3* gene expression by CircHIPK3 promotes proliferation and invasion in nasopharyngeal carcinoma which affect the prognosis of patients [23]. Similarly, Zhao W et al. reported that *ELF3* regulates cell metastasis and invasion in non-small cell lung cancer via the *PI3K/Akt* pathway, thereby affecting prognosis of patients [24]. In view of the studies mentioned above, this is the first time *ELF3* has been reported to affect the prognosis of patients with TETs, which suggest differences in the pathogenesis of TETs compared to other tumors. *ILRN* gene is mainly involved in immune response and is regulated by the cg02543462 site [25]. *ILRN* involvement in immune response and TETs association with autoimmune disorders [26] may suggest a possible mechanism on how *ILRN* could affect the prognosis of patients with TETs. The *RAG1* is mainly involved in immune regulation and is regulated by the cg06288355 site [27]. Results from this study showed that both *ILRN* and *RAG1* genes affected the prognosis of patients with TETs, possibly through the immune regulation of TETs. *KSR1*, *ELF3*, *ILRN*, and *RAG1* regulated by the DNA methylation sites cg05784862, cg07154254, cg02543462, and cg06288355, respectively, are known to be involved in tumorigenesis, progression, and death, which is one of the reasons for using them as prognostic biomarkers in patients with TETs.

Currently, Masaoka staging is commonly used for the prognosis of TETs, however, the accuracy of Masaoka staging needs to be improved. Therefore, it is important to search for new biomarkers in order to understand the prognosis of patients with TETs. Previous study by Wei J et al. had collected and analyzed the miRNA expression of patients with TETs from the TCGA data portal. The results showed that seven miRNAs were associated with the overall survival in patients with TETs and could be used as a differential diagnosis for type C TETs [28]. Likewise, the differential DNA methylation sites found in this study were able to determine the prognosis of patients with TETs and has higher accuracy than the commonly used Masaoka staging system. In addition, these sites can also differentially diagnose type C TETs. The above results were further validated by sequencing our own specimen. Santoni G et al. reported that high *CTLA-4* protein expression correlates with poor prognosis of patients with TETs, suggesting that *CTLA-4* can be used as a prognostic factor in TETs [29]. However, the drawback is that there is no comparison with the current Masaoka staging system. Literature had also reported that overexpression of focal adhesion kinase (*FAK*) protein in TETs was associated with poor prognosis and may serve as an independent prognostic biomarker for TETs [30]. Xu RH et al. reported that DNA methylation sites can be used for tumor diagnosis and prognosis, which further support our current findings [31]. At present, tumor research has entered the era of omics; it is difficult for a single gene or protein to determine the prognosis of tumor patients. Therefore, several DNA methylation sites that affect the prognosis of patients with TETs were proposed, forming a prognostic model which further enhance the accuracy in determining the prognosis and is also more accurate than the current Masaoka staging system.

Although a prognostic model for TETs was successfully constructed using DNA methylation sites, the number of cases can still be improved, and more experiments are needed to further validate these results. Currently, the mechanism of *KSR1*, *ELF3*, *ILRN*, and *RAG1* in regulating the prognosis of patients with TETs is not fully understood. Therefore, more experiments are needed to understand these mechanisms.

## Conclusions

Taken together, *ELF3*, *KSR1*, *ILRN*, and *RAG1* methylation sites can be used to determine the prognosis of patients with TETs and can also differentially diagnose subtypes of TETs. This combination of methylation sites can guide clinical diagnosis in differentiating thymoma and thymic carcinoma and better determine the prognosis of patients.

## Methods

### TCGA thymoma datasets

The preprocessed methylation dataset of thymoma TCGA.THYM.sampleMap/Human Methylation450(v.2017-09-08) was downloaded from UCSC (https://xenabrowser.net/ datapages/). This dataset contains DNA methylation profiles of 124 tumor tissues and 2 matched adjacent normal tissues from 124 cases of thymoma as discovery set. DNA methylation profile was measured experimentally using the Illumina Infinium HumanMethylation450 platform. Microarray probes are mapped onto the human genome coordinates using xena probeMap derived from GEO GPL13534 record (https://www.ncbi.nlm.nih.gov/geo/query/acc.cgi?acc=GPL13534). *M* values obtained from logarithm transformation from beta values were used for statistical analyses and beta-values were used for heatmap visualizations and clustering [32]. A raw counts matrix of gene-level RSEM values from 122 cases of thymoma was obtained from http://gdac.broadinstitute.org/runs/stddata__2015_11_01/data/THYM/20151101/. This dataset was used to identify differentially expressed genes between groups which consisted of thymoma type A to B3 as well as type C thymic carcinoma. Clinical information of this TCGA cohort such as WHO histological type and Masaoka stage was also obtained from UCSC (TCGA.THYM.sampleMap/THYM_clinicalMatrix, v.2016-04-27).

#### Differential methylation analysis, differential expression analysis, and identification of epigenetically regulated genes in discovery set

DNA methylation probes with at least one “NA” across all samples were removed, and 392,653 probes were left for further analysis. To explore the thymic carcinoma-specific DNA hypermethylation or hypomethylation events, two sample *t* test was used to perform site-level testing between thymoma type A to B3 (*n* = 113) and type C thymic carcinoma (*n* = 11) using transformed *M* values and Bonferroni procedure was used to adjust crude *P* values for multiple comparisons. All probes with adjusted *P* < 0.05 and estimated mean difference of methylation between thymoma type A to B3 and type C thymic carcinoma of at least 50% were considered as candidate methylation sites for further identification of epigenetically regulated genes. Probes that were located within a promotor region were identified using GEO GPL13534 annotation file. Differential expression analysis between thymoma type A to B3 and type C thymic carcinoma was carried out with R/Bioconductor limma package after filtering genes with very low counts and the voom transformation. FDR < 0.05 and |log2Ratio| ≥ 1 was set as the threshold for significantly differential expression. DNA methylation and gene expression data were merged by gene symbol. Genes that met the following criteria were considered epigenetically silenced or upregulated respectively: △β > 50% and log2Ratio < − 1 or △β < − 50% and log2Ratio > 1.

### Unsupervised clustering analysis

A total of 694 methylation sites within the promotor region that could potentially regulate gene expression were identified through the procedure described above. The beta values of thymic carcinoma-specific DNA methylation sites were used to perform unsupervised hierarchical clustering on all 124 cases of thymoma using the Cluster 3.0 program in the Biopython package [33] and visualized using JAVA TreeView program [34]. All pairwise distance between the patients was measured with Euclidean distance and pairwise maximum linkage clustering was used to define the distance between clusters.

### Survival analysis for identification of candidate methylation sites responsible for overall survival prognosis

Survival analysis was carried out using Cox proportional hazards model as implemented in R survival package against overall survival data in discovery set. Univariate and multiple variable Cox regression were separately used to evaluate the prognosis value of 694 individual probes identified as epigenetically regulated sites. Beta values were represented by continuous variables and Masaoka stage, whereas WHO histological type was represented by binary variables. During adjustment for two important clinical prognostic factors in multiple variables Cox regression, Masaoka stages I and II were categorized into one state and Masaoka stages III and IV into another state. WHO histological type A to B3 was grouped into one state and type C into another state. The likelihood ratio test was used to determine if beta value from one probe entered into the regression models that contains Masaoka stage and WHO histological type as covariates is significant. Only probes with crude *P* value <  0.05 in both univariate and multiple variable Cox regression were considered statistically significant and were identified as candidate probes for further validation for overall survival prognosis.

### Patients for validation

To verify the association of the methylation status of these four candidate genes with overall survival, a total of 100 patients with histologically confirmed thymoma or thymic carcinoma who were admitted into the Department of Thoracic Surgery at Daping hospital of the Third Military Medical University, China between October 2007 and October 2017 were enrolled in the study. Patients with concomitant malignant neoplasms were excluded. Tumors were reviewed and reclassified according to the 2015 WHO criteria. Tumor staging was performed according to the revised Masaoka system. This was used as the validation set. This study was approved by the Medical Ethics Committee at our hospital. Written informed consent was obtained from all patients prior to their enrolment. Patient information including age, sex, histology, presence of myasthenia gravis, Masaoka stage, WHO histological type and follow-up information including the follow-up period, time of the last follow-up, and overall survival of patients were obtained from clinical records and questionnaires.

### Pyrosequencing

Resected specimens were obtained via complete tumor resection, fixed with 10% formalin, embedded in paraffin, and divided into 10 μm sections. Genomic DNA was extracted from 10 sections using the QIAamp DNA FFPE Tissue kit (Qiagen, Hilden, Germany). The concentration and purity of these DNA samples were determined with a spectrophotometer (NanoDrop2000, Thermo Scientific, Waltham, MA, USA). Bisulfite conversion of total 500 ng purified DNA in each sample was performed with EZ DNA Methylation-GoldTM Kit according to manufacturer’s instructions (Cat. No. D5006, Zymo Research Corporation, Orange, CA, USA). The bisulfite conversed DNA was amplified with TaKaRa EpiTaqTM HS (Cat. No. R110A, Takara Biomedical Technology (Beijing) Co., Ltd. Beijing, China) with reaction setup: 10 ng bisulfite-treated DNA, 0.4 μM forward and reverse primers, 2.5 μL 10 × EpiTap PCR Buffer, 2.5 mM MgCl2, dNTP Mixture (0.264 mM each), EpiTap HS(0.025 U/μL) in total 25 μL each reaction and with following thermal cycle condition: denaturation at 98 °C for 10 s, annealing at 55 °C for 30 s, extension at 72 °C for 30 s executed for 35 cycles followed by extension at 72 °C for 1 min and hold at 4 °C. The amplicons were then subjected to pyrosequencing with PyroMark Q96 (Qiagen, Hilden, Germany). All primers used are presented in Additional file 8: Table S1.

### Statistical analysis

All beta values and mRNA expression levels were represented with median value, range, and visualized with a box plot. The difference in four methylation sites between thymoma type A to B3 and type C thymic carcinoma and the difference in mRNA expression levels between low and high methylation subgroups was evaluated by Kruskal-Wallis test. Kaplan-Meier method and the log-rank test were used to compare the overall survival between low and high methylation subgroups. A weighted model was constructed for prognostic model [35]. The total sum of products with coefficient of four candidate methylation sites in univariate Cox regression and corresponding beta values were calculated as risk scores for each patient in the validation set. The predictive efficiency of risk score, Masaoka stage, and methylation in cg05784862(*KSR1*) for 5-year overall survival in the validation set was determined with time-dependent ROC curve analysis using function “timeROC.” Comparison between two time-dependent AUCs was performed with function “compare” embedded in R language package “timeROC” (version 0.3 published in 2015-03-25) [36]. All other statistical analyses were performed using SPSS 17.0 (IBM SPSS, Chicago, IL, USA). All tests were bilateral, and *P* < 0.05 was considered statistically significant.

## Additional files

Additional file 1: **Table S2.** List of significantly expressed methylation probes. In this table, the “mean_AB” and “mean_C” stands for the average values of beta values in cases with WHO histological type A to B3 and WHO histological type C, respectively. “FC” for fold changes which is calculated as (mean_C- mean_AB)/mean_AB. The “p” for crude probabilities resulting from t test for each probe. (XLSX 1490 kb)

Additional file 2: **Table S3.** Results from KEGG enrichment analysis for genes corresponding to the significantly expressed methylation probes. (XLSX 17 kb)

Additional file 3: **Table S4.** The list of genes transcriptionally affected by methylation of significantly expressed methylation probes within promotor regions. In this table, meaning of the “mean_AB”, “mean_C”, “FC” and “p” is the same asdescribed in “Additional file 1: Table S2”. (XLSX 119 kb)

Additional file 4: **Figure S1.** A heatmap showing methylation profiles of 542 significantly expressed methylation sites which localize within promotor regions in corresponding genes and could be involved in regulation of mRNA expression for genes across patients with WHO histological type A to B3. (PNG 93 kb)

Additional file 5: **Table S5.** Summary of results from univariate and multivariable Cox regression for overall survival of probes which potentially affect transcription mRNA. The “Pcg” in this table respectively stands for probabilities for methylation of each probe adjusted for WHO histological type, Masaoka stage based on likelihood ratio test. The “Pt” for probabilities of significance of main effect model including WHO histological type, Masaoka stage and methylation of each probe. The “coeff” for coefficients of each probe in multivariable Cox regression. (XLSX 63 kb)

Additional file 6: **Figures S2.** Box plots showing the distribution of beta values in cg05784862(KSR1), cg07154254(ELF3), cg02543462(ILRN) and cg06288355(RAG1) between thymoma patients with WHO histological type C and type A to B3 in TCGA dataset. (TIFF 403 kb)

Additional file 7: **Figures S3.** Scatter plots showing relationship between mRNA expression levels and methylation in four candidate methylation sites cg05784862(KSR1), cg07154254(ELF3), cg02543462(ILRN) and cg06288355(RAG1). The red dots in each square indicate cases with WHO histological type C. (TIFF 734 kb)

Additional file 8: **Table S1.** List of primers used for pyrosequencing and quantitative RT-PCR. (XLSX 10 kb)

## Abbreviations

*ELF3*: E74 like ETS transcription factor 3
*ILRN*: Interleukin 1 receptor type 1
KEGG: Kyoto Encyclopedia of Genes and Genomes
*KSR*: Kinase suppressor of ras 1
*RAG1*: Recombination activating 1
ROC: Receiver operating characteristic curve
TETs: Thymic epithelial tumors

## References

[1] Engels EA (2010). Epidemiology of thymoma and associated malignancies. J Thorac Oncol.

[2] Travis WD, Brambilla E, Burke AP, Marx A, Nicholson AG (2015). Introduction to the 2015 World Health Organization classification of tumors of the lung, pleura, Thymus, and heart. J Thorac Oncol.

[3] Kelly RJ, Petrini I, Rajan A, Wang Y, Giaccone G (2011). Thymic malignancies: from clinical management to targeted therapies. J Clin Oncol.

[4] Kondo K, Yoshizawa K, Tsuyuguchi M, Kimura S, Sumitomo M, Morita J, Miyoshi T, Sakiyama S, Mukai K, Monden Y (2004). WHO histologic classification is a prognostic indicator in thymoma. Ann Thorac Surg.

[5] Feng Y, Lei Y, Wu X, Huang Y, Rao H, Zhang Y, Wang F (2017). GTF2I mutation frequently occurs in more indolent thymic epithelial tumors and predicts better prognosis. Lung Cancer.

[6] Hishida T, Nomura S, Yano M, Asamura H, Yamashita M, Ohde Y, Kondo K, Date H, Okumura M, Nagai K (2016). Japanese Association for Research on the Thymus (JART). Long-term outcome and prognostic factors of surgically treated thymic carcinoma: results of 306 cases from a Japanese Nationwide database study. Eur J Cardiothorac Surg.

[7] Marx A, Chan JK, Coindre JM, Detterbeck F, Girard N, Harris NL, Jaffe ES, Kurrer MO, Marom EM, Moreira AL, Mukai K, Orazi A, Ströbel P (2015). The 2015 World Health Organization classification of tumors of the thymus: continuity and changes. J Thorac Oncol.

[8] Kajiura K, Takizawa H, Morimoto Y, Masuda K, Tsuboi M, Kishibuchi R, Wusiman N, Sawada T, Kawakita N, Toba H, Yoshida M, Kawakami Y, Naruto T, Imoto I, Tangoku A, Kondo K (2017). Frequent silencing of RASSF1A by DNA methylation in thymic neuroendocrine tumours. Lung Cancer.

[9] Gong M, Shi W, Qi J, Shao G, Shi Z, Wang J, Chen J, Chu R (2017). Alu hypomethylation and MGMT hypermethylation in serum as biomarkers of glioma. Oncotarget.

[10] Kont V, Murumägi A, Tykocinski LO, Kinkel SA, Webster KE, Kisand K, Tserel L, Pihlap M, Ströbel P, Scott HS, Marx A, Kyewski B, Peterson P (2011). DNA methylation signatures of the AIRE promoter in thymic epithelial cells, thymomas and normal tissues. Mol Immunol.

[11] Owen D, Chu B, Lehman AM, Annamalai L, Yearley JH, Shilo K, Otterson GA (2018). Expression patterns, prognostic value, and Intratumoral heterogeneity of PD-L1 and PD-1 in thymoma and thymic carcinoma. J Thorac Oncol.

[12] Kim BS, Kim JK, Kang CH, Kim YT, Jung KC, Won JK (2018). An immunohistochemical panel consisting of EZH2, C-KIT, and CD205 is useful for distinguishing thymic squamous cell carcinoma from type B3 thymoma. Pathol Res Pract.

[13] Fukui T, Fukumoto K, Okasaka T, Kawaguchi K, Nakamura S, Hakiri S, Ozeki N, Hirakawa A, Tateyama H, Yokoi K (2016). Prognostic impact of tumour size in completely resected thymic epithelial tumours. Eur J Cardiothorac Surg.

[14] Ren XY, Wen X, Li YQ, Zhang J, He QM, Yang XJ, Tang XR, Wang YQ, Zhang PP, Chen XZ, Cheng B, Ma J, Liu N (2018). TIPE3 hypermethylation correlates with worse prognosis and promotes tumor progression in nasopharyngeal carcinoma. J Exp Clin Cancer Res.

[15] Merkerova MD, Remesova H, Krejcik Z, Loudova N, Hrustincova A, Szikszai K, Cermak J, Jonasova A, Belickova M (2018). Relationship between altered miRNA expression and DNA methylation of the DLK1-DIO3 region in azacitidine-treated patients with myelodysplastic syndromes and acute myeloid leukemia with myelodysplasia-related changes. Cell.

[16] Radovich M, Pickering CR, Felau I, Ha G, Zhang H, Jo H, Hoadley KA, Anur P, Zhang J, McLellan M, Bowlby R, Matthew T, Danilova L, Hegde AM, Kim J, Leiserson MDM, Sethi G, Lu C, Ryan M, Su X, Cherniack AD, Robertson G, Akbani R, Spellman P, Weinstein JN, Hayes DN, Raphael B, Lichtenberg T, Leraas K, Zenklusen JC, Cancer genome atlas network, Fujimoto J, Scapulatempo-Neto C, Moreira AL, Hwang D, Huang J, Marino M, Korst R, Giaccone G, Gokmen-Polar Y, Badve S, Rajan A, Ströbel P, Girard N, Tsao MS, Marx A, Tsao AS, Loehrer PJ (2018). The integrated genomic landscape of thymic epithelial tumors. Cancer Cell.

[17] Chen Y, Zhang Y, Chai X, Gao J, Chen G, Zhang W, Zhang Y (2018). Correlation between the expression of PD-L1 and clinicopathological features in patients with thymic epithelial tumors. Biomed Res Int.

[18] Merveilleux du Vignaux C, Dansin E, Mhanna L, Greillier L, Pichon E, M K, Clément-Duchêne C, Mennecier B, Westeel V, Robert M, Quantin X, Zalcman G, Thiberville L, Lena H, Molina T, Calcagno F, Fournel P, Mazières J, Besse B, Girard N (2018). Systemic therapy in advanced thymic epithelial tumors: insights from the RYTHMIC prospective cohort. J Thorac Oncol.

[19] Stevens PD, Wen YA, Xiong X, Zaytseva YY, Li AT, Wang C, Stevens AT, Farmer TN, T G, Weiss HL, Inagaki M, Marchetto S, Borg JP, Gao T (2018). Erbin suppresses KSR1-mediated RAS/RAF signaling and tumorigenesis in colorectal Cancer. Cancer Res.

[20] McCall JL, Gehring D, Clymer BK, Fisher KW, Das B, Kelly DL, Kim H, White MA, Lewis RE (2016). KSR1 and EPHB4 regulate Myc and PGC1β to promote survival of human colon tumors. Mol Cell Biol.

[21] Neilsen BK, Frodyma DE, Lewis RE, Fisher KW (2017). KSR as a therapeutic target for Ras-dependent cancers. Expert Opin Ther Targets.

[22] Luk IY, Reehorst CM, Mariadason JM (2018). ELF3, ELF5, EHF and SPDEF transcription factors in tissue homeostasis and cancer. Molecules.

[23] Ke Z, Xie F, Zheng C, Chen D (2019). CircHIPK3 promotes proliferation and invasion in nasopharyngeal carcinoma by abrogating miR-4288-induced ELF3 inhibition. J Cell Physiol.

[24] Zhao W, Sun Q, Yu Z, Mao S, Jin Y, Li J, Jiang Z, Zhang Y, Chen M, Chen P, Chen D, Xu H, Ding S, Yu Z (2018). MiR-320a-3p/ELF3 axis regulates cell metastasis and invasion in non-small cell lung cancer via PI3K/Akt pathway. Gene.

[25] Saitoh M, Kobayashi K, Ohmori I, Tanaka Y, Tanaka K, Inoue T, Horino A, Ohmura K, Kumakura A, Takei Y, Hirabayashi S, Kajimoto M, Uchida T, Yamazaki S, Shiihara T, Kumagai T, Kasai M, Terashima H, Kubota M, Mizuguchi M (2016). Cytokine-related and sodium channel polymorphism as candidate predisposing factors for childhood encephalopathy FIRES/AERRPS. J Neurol Sci.

[26] Oji Y, Inoue M, Takeda Y, Hosen N, Shintani Y, Kawakami M, Harada T, Murakami Y, Iwai M, Fukuda M, Nishida S, Nakata J, Nakae Y, Takashima S, Shirakata T, Nakajima H, Hasegawa K, Kida H, Kijima T, Morimoto S, Fujiki F, Tsuboi A, Morii E, Morita S, Sakamoto J, Kumanogoh A, Y O, Okumura M, Sugiyama H (2018). WT1 peptide-based immunotherapy for advanced thymic epithelial malignancies. Int J Cancer.

[27] Abd Hamid IJ, Slatter MA, McKendrick F, Pearce MS, Gennery AR (2018). Long-term health outcome and quality of life post-HSCT for IL7Rα-, Artemis-, RAG1- and RAG2-deficient severe combined immunodeficiency: a single center report. J Clin Immunol.

[28] Wei J, Liu Z, Wu K, Yang D, He Y, Chen GG, Zhang J, Lin J (2017). Identification of prognostic and subtype-specific potential miRNAs in thymoma. Epigenomics.

[29] Santoni G, Amantini C, Morelli MB, Tomassoni D, Santoni M, Marinelli O, Nabissi M, Cardinali C, Paolucci V, Torniai M, Rinaldi S, Morgese F, Bernardini G, Berardi R (2018). High CTLA-4 expression correlates with poor prognosis in thymoma patients. Oncotarget.

[30] Li M, Hou F, Zhao J, Zhang T, Li D, Wu W, Liu X (2018). Xu L. focal adhesion kinase is overexpressed in thymic epithelial tumors and may serve as an independent prognostic biomarker. Oncol Lett.

[31] Xu RH, Wei W, Krawczyk M, Wang W, Luo H, Flagg K, Yi S, Shi W, Quan Q, Li K, Zheng L, Zhang H, Caughey BA, Zhao Q, Hou J, Zhang R, Xu Y, Cai H, Li G, Hou R, Zhong Z, Lin D, Fu X, Zhu J, Duan Y, Yu M, Ying B, Zhang W, Wang J, Zhang E, Zhang C, Li O, Guo R, Carter H, Zhu JK, Hao X, Zhang K (2017). Circulating tumour DNA methylation markers for diagnosis and prognosis of hepatocellular carcinoma. Nat Mater.

[32] Du P, Zhang X, Huang CC, Jafari N, Kibbe WA, Hou L, Lin SM (2010). Comparison of Beta-value and M-value methods for quantifying methylation levels by microarray analysis. BMC Bioinformatics.

[33] de Hoon MJ, Imoto S, Nolan J, Miyano S (2004). Open source clustering software. Bioinformatics.

[34] Saldanha AJ (2004). Java Treeview--extensible visualization of microarray data. Bioinformatics.

[35] Li YQ, Tian YM, Tan SH, Liu MZ, Kusumawidjaja G, Ong EHW, Zhao C, Tan TWK, Fong KW, Sommat K, Soong YL, Wee JTS, Han F, Chua MLK (2018). Prognostic model for stratification of radioresistant nasopharynx carcinoma to curative salvage radiotherapy. J Clin Oncol.

[36] Blanche P, Dartigues J-F, Jacqmin-Gadda H (2013). Estimating and comparing time-dependent areas under receiver operating characteristic curves for censored event times with competing risks. Stat Med.

