# Supplementary figures and images for: Discovery and validation of DNA methylation markers for overall survival prognosis in patients with thymic epithelial tumors
Source: Clin Epigenetics. 2019 Mar 4;11:38. doi: 10.1186/s13148-019-0619-z (PMC6398263; doi:10.1186/s13148-019-0619-z)

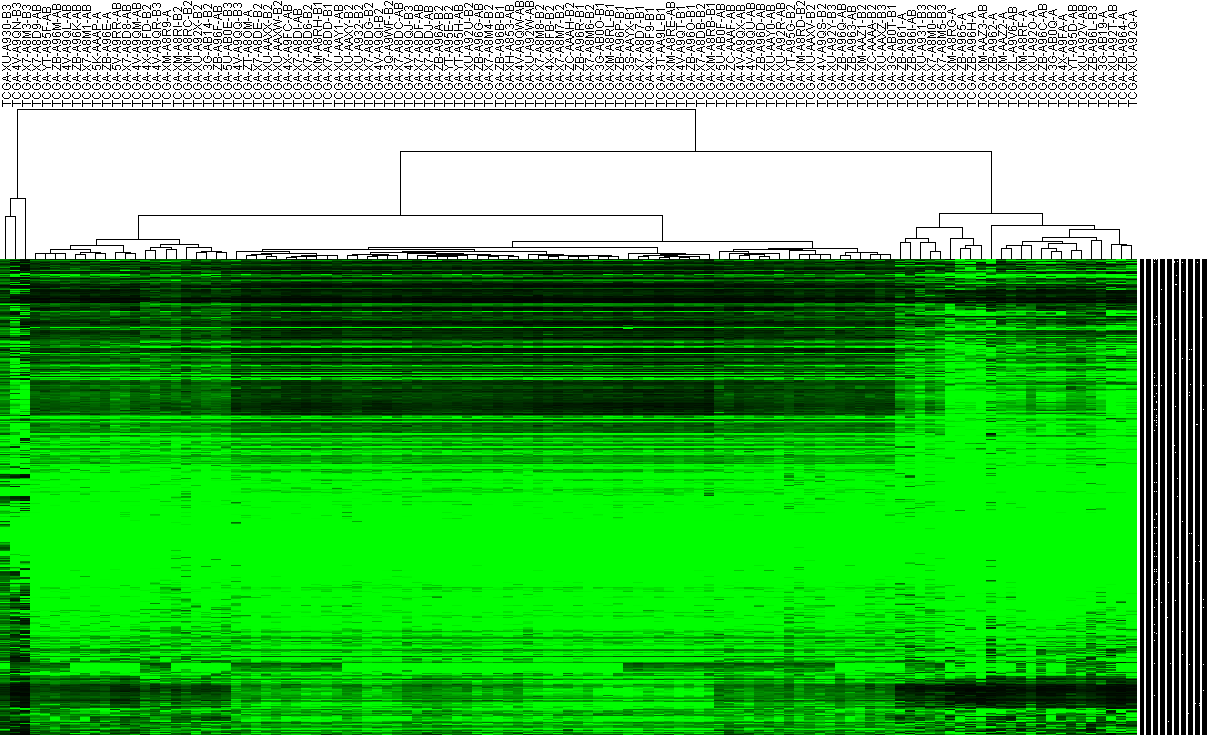

Supplement: Supplementary file 4 — Figure S1. A heatmap showing methylation profiles of 542 significantly expressed methylation sites which localize within promotor regions in corresponding genes and could be involved in regulation of mRNA expression for genes across patients with WHO histological type A to B3. (PNG 93 kb) [file 13148_2019_619_MOESM4_ESM.png]

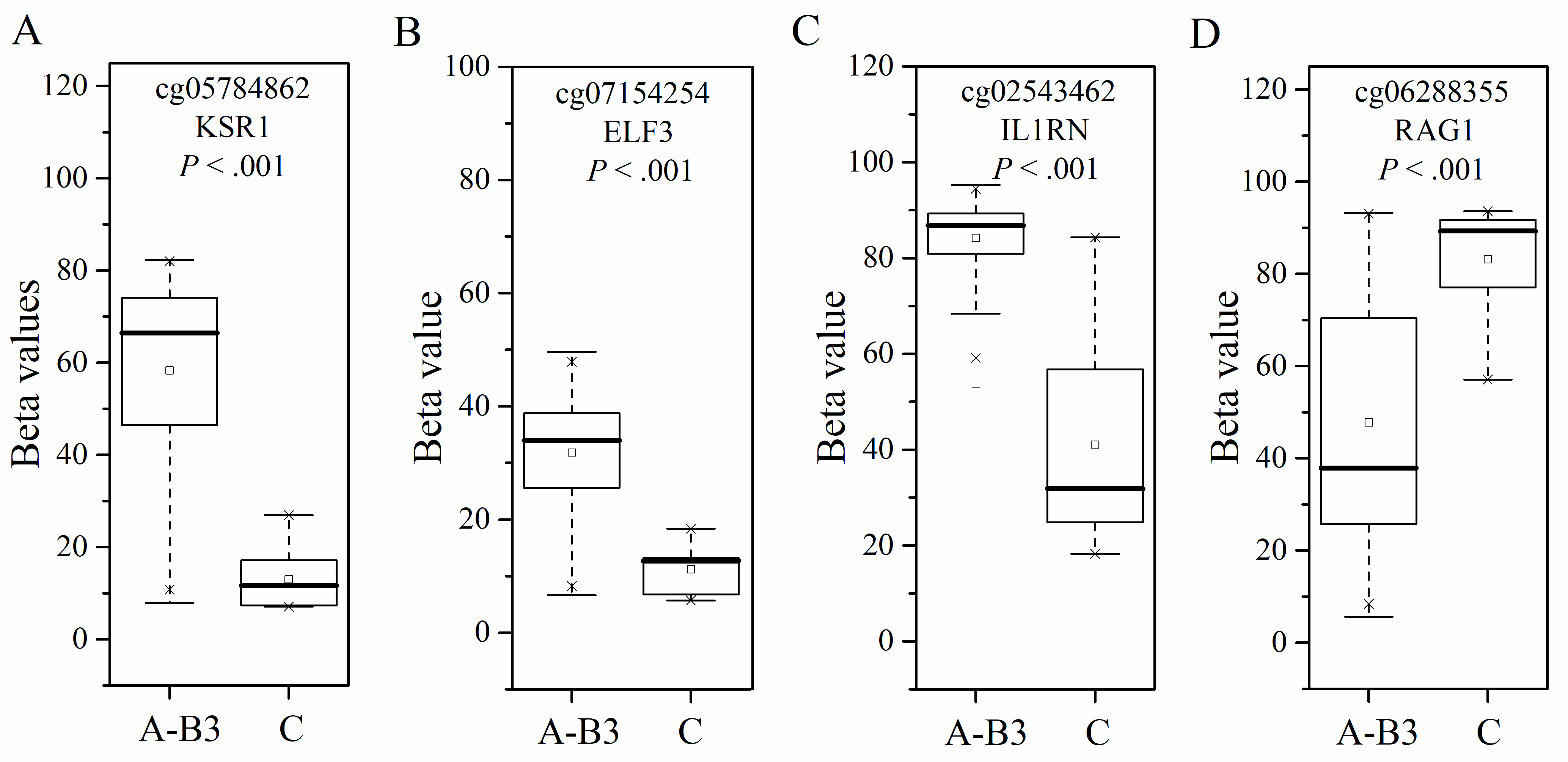

Supplement: Supplementary file 6 — Figures S2. Box plots showing the distribution of beta values in cg05784862(KSR1), cg07154254(ELF3), cg02543462(ILRN) and cg06288355(RAG1) between thymoma patients with WHO histological type C and type A to B3 in TCGA dataset. (TIFF 403 kb) [file 13148_2019_619_MOESM6_ESM.tif]

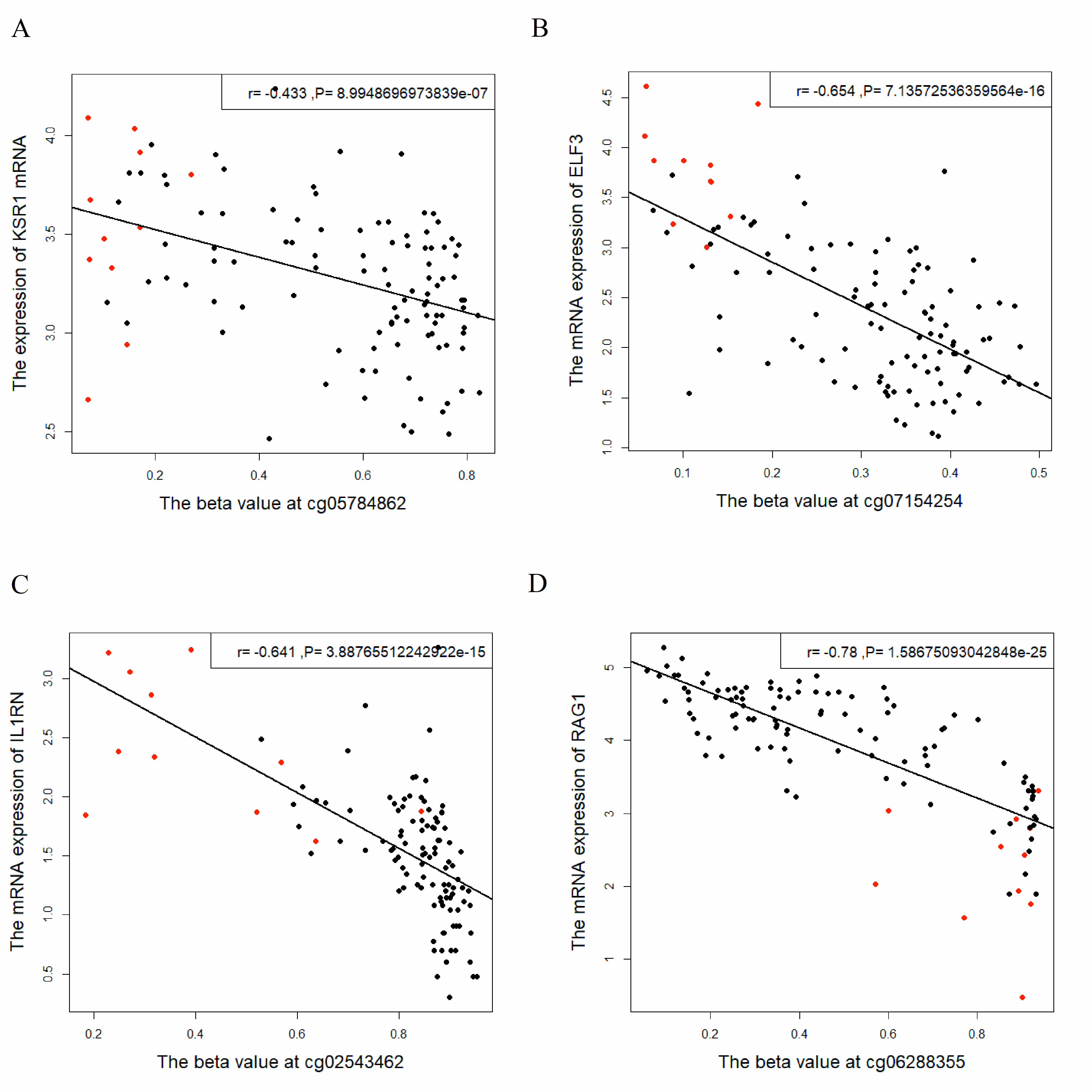

Supplement: Supplementary file 7 — Figures S3. Scatter plots showing relationship between mRNA expression levels and methylation in four candidate methylation sites cg05784862(KSR1), cg07154254(ELF3), cg02543462(ILRN) and cg06288355(RAG1). The red dots in each square indicate cases with WHO histological type C. (TIFF 734 kb) [file 13148_2019_619_MOESM7_ESM.tif]
